# Field recommended concentrations of pyraclostrobin exposure disturb the development and immune response of worker bees (*Apis mellifera* L.) larvae and pupae

**DOI:** 10.3389/fphys.2023.1137264

**Published:** 2023-02-09

**Authors:** Manqiong Xiong, Gan Qin, Lizhu Wang, Ruyi Wang, Ruiqi Zhou, Xiaotian Luo, Qun Lou, Shaokang Huang, Jianghong Li, Xinle Duan

**Affiliations:** ^1^ College of Animal Science (College of Bee Science), Fujian Agriculture and Forestry University, Fuzhou, China; ^2^ Fujian Honey Bee Biology Observation Station, Ministry of Agriculture and Rural Affairs, Fuzhou, China

**Keywords:** *Apis mellifera*, pyraclostrobin, larvae, pupae, gene expression

## Abstract

The strobilurin fungicide pyraclostrobin is widely used to prevent and control the fungal diseases of various nectar and pollen plants. Honeybees also directly or indirectly contact this fungicide with a long-term exposure period. However, the effects of pyraclostrobin on the development and physiology of *Apis mellifera* larvae and pupae during continuous exposure have been rarely known. To investigate the effects of field-realistic concentrations of pyraclostrobin on honeybee survival and development, the 2-day-old larvae were continuously fed with different pyraclostrobin solutions (100 mg/L and 83.3 mg/L), and the expression of development-, nutrient-, and immune-related genes in larvae and pupae were examined. The results showed that two field-realistic concentrations of pyraclostrobin (100 and 83.3 mg/L) significantly decreased the survival and capped rate of larvae, the weight of pupae and newly emerged adults, and such decrease was a positive correlation to the treatment concentrations. qPCR results showed that pyraclostrobin could induce the expression of Usp, ILP2, Vg, Defensin1, and Hymenoptaecin, decrease the expression of Hex100, Apidaecin, and Abaecin in larvae, could increase the expression of Ecr, Usp, Hex70b, Vg, Apidaecin, and Hymenoptaecin, and decreased the expression of ILP1, Hex100 and Defensin1in pupae. These results reflect pyraclostrobin could decrease nutrient metabolism, immune competence and seriously affect the development of honeybees. It should be used cautiously in agricultural practices, especially in the process of bee pollination.

## 1 Introduction

The honeybee is the most important economic insect. It is not only the ideal pollinator of crops and wild plants that subserve the agricultural production, plant diversity and ecological balance but also provides nutritious bee products (Gallai et al., 2009; Champetier et al., 2015; Zheng et al., 2019). In recent years, the honeybee colony losses were reported and the dramatic reductions caused significant economic losses all over the world (Van der Zee et al., 2012; van Dooremalen et al., 2018; Calatayud-Vernich et al., 2019; Kablau et al., 2020). The continued decline of honeybee colonies brings a serious crisis to plant pollination and food production, because nearly 75% of the major crop species rely on pollinators (Clermont et al., 2015; Shi et al., 2020). The main reasons were diverse agrochemicals, parasites, viruses, adjustment of crop planting structure and distribution, especially the noticeable toxic effects of pesticides on honeybees, and also these factors were complex and interacting (van Dooremalen et al., 2018; Duan et al., 2020a; Shi et al., 2020b). During the foraging process, honeybees can directly contact the pesticides remaining on the surface of plants (Legard et al., 2001; Xiong et al., 2022). At the same time, the systemic pesticide residues could be absorbed by plants and remain in nectar and pollen, then were taken back to the colony by the foraging bee and led to the contamination of honey, pollen and comb which were consumed by other members in the colony (Mullin et al., 2010). As a metamorphosis development insect, the life cycle of the honeybee consists of four developmental phases, including egg, larva, pupa, and adult. Furthermore, these pesticide residues could harm the health of honeybees at different stages of individuals and colonies by direct or indirect exposure (Krupke et al., 2017). Consequently, the primary problem in modern agricultural production is how to balance between protecting crops efficiently against pests and diseases and maintaining healthy pollinator populations (Godfray and Garnett, 2014; Xiong et al., 2022).

As an important class of plant protection products, fungicides have already accounted for more than 35% of the global pesticide market and are widely used in the disease control of nectar and pollen plants, such as maize, rape, sunflower, and alfalfa, which account for approximately 11% of total global pesticide use (Liao et al., 2018; Zhang 2018; Zubrod et al., 2019). According to the data of the Fungicide Resistance Action Committee (FRAC), the action mechanisms of fungicides to plant pathogenic microorganisms were the negative effects on the nucleic acid and protein synthesis, respiration, signal transduction, cell division, and membrane structure and function of microorganisms (Fungicide Resistance Action Committee, 2021). Therefore, the bioassay results showed that most fungicides were lower acutely toxic to honeybees and other non-target insects (Liao et al., 2018; Simondelso et al., 2018; Xiong et al., 2022). Normally, the field-realistic concentrations or residue in the colonies were considered to be low toxicity to cause illness or death of honeybees (Pettis et al., 2013). Furthermore, the fungicides were considered to be safe for honeybees and the chronic toxicity was ignored (Tadei et al., 2019; Duan et al., 2020a; Xiong et al., 2022). Thus bees are more likely to encounter fungicides than insecticides because fungicides can even be sprayed when insect-attractive crops are in bloom (Favaro et al., 2019; Gierer et al., 2019; Rondeau and Raine 2022).

However, a large number of scientific investigations have reported the chronic toxicity of fungicides on the development, detoxification, and immune function, foraging, and homing ability, olfactory memory of bees, which cause serious damage to the individual and colony of honeybees (Zhu et al., 2014; Carneiro et al., 2019; Duan et al., 2020a; Dai et al., 2021; Fisher et al., 2021; Traynor et al., 2021). After exposure to chlorothalonil for 3 days, the mortality of *A. mellifera* 4-day-old larvae was significantly increased over two-fold compared to untreated larvae, and also the pairing of chlorothalonil and coumaphos or fluvalinate produced synergistic interactions on the mortality of larvae (Zhu et al., 2014). The low field concentration of dimertachlone, prochloraz and iprodione could induce the activities of catalase (CAT), carboxylesterases (CarE) and glutathione S-transferase (GSTs), but the high concentration inhibits their activity (Duan et al., 2020a). The Iprodione (2 mg/kg) was not lethal to newly emerged bees, but it can inhibit the synthesis of glutathione, leading to the generation of reactive oxygen species and the cells of treated bees had signs of apoptosis (Carneiro et al., 2019). The benomyl stress (5 g/kg) led to a total of 5,759 DEGs being upregulated in the worker bees of *A. mellifera*, and most of the DEGs were involved in the functions of immunity, detoxification, biological metabolism, and regulation, such as light conduction, MAPK, calcium ion pathway and other 12 pathways (Dai et al., 2021). DesJardins et al. (2021) found the compound fungicides Pristine^®^ showed significant sublethal effects on the learning performance of *A. mellifera* and lead the work type conversion of nurse bee to forage bee.

Pyraclostrobin is a high-efficiency, low-toxic, and broad-spectrum systemic strobilurin fungicide which was registered and widely used to prevent and control diseases caused by fungi on various nectar and pollen plants (Bartlett et al., 2002). The bactericidal mechanism of pyraclostrobin was to inhibit cell respiration in fungi and the acute oral and contact toxicity of pyraclostrobin to the worker bee of *A. mellifera* was low toxicity (LC_50_ > 100 μg (a.i.)/bee) (Earley et al., 2012; Tan et al., 2021). Pyraclostrobin was chronic toxicity to honeybees and could directly inhibit the mitochondrial function *in vitro* (Campbell et al., 2016; Nicodemo et al., 2020). The field-relevant doses of pyraclostrobin decreased the height of secretory cells and volume of mandibular glands with 6 days continuous exposure and influence the behavior of newly emerged workers and young workers (Zaluski et al., 2017; Tadei et al., 2019). Meanwhile, pyraclostrobin was widely residual in the pollen of treated crops and in honeybee colonies which may influence the health of honeybees (Yoder et al., 2013; David et al., 2015).

However, the effect of pyraclostrobin on the development and physiology of larvae and pupae of *A. mellifera* is rarely known. To investigate the influence of field-realistic concentrations of pyraclostrobin on larvae and pupae, the survival and developmental state of *A. mellifera* worker bees from larvae to adult stage in each treatment were documented daily. Further, the effect of pyraclostrobin on the development-related genes ecdysone receptor (*Ecr*) and ultraspiracle protein (*Usp*), nutrient metabolism-related genes insulin-like peptides 1 (*ILP 1*), insulin-like peptides 2 (*ILP 2*), *Hexamerin 70b* (*Hex 70b*), *Hexamerin 110* (*Hex 110*), *Vitellogenin* (*Vg*) and immune-related genes *Apidaecin*, *Abaecin*, *Hymenoptaecin*, *Defensin1* in larvae and pupae were examined, respectively. This study will provide new evidence of pyraclostrobin exposure on honeybee larvae and pupae development, and also provide the theoretical basis for the pollination safety and the management of pesticides.

## 2 Materials and methods

### 2.1 The fungicide and treated concentrations

The 25% pyraclostrobin suspension concentrate was purchased from Hebei Chengyue Chemical Co., Ltd. According to the fungicide instruction manual, the recommended dilution multiple of pyraclostrobin for disease control was 2500–3000. Considering the actual use of the field, two field-realistic concentrations of pyraclostrobin 100 mg/L (2500 fold) and 83.3 mg/L (3000 fold) were designed and diluted by the artificial diet of larvae, which were stored at −4°C and used up within 7 days. Different day-old larvae have different artificial diets which should be prepared when using. Diet A for 1 and 2 day-old larvae (royal jelly 50%, glucose 6%, fructose 6%, yeast extract 1%, and water 37%), Diet B for 3 day-old larvae (royal jelly 50%, glucose 7.5%, fructose 7.5%, yeast extract 1.5%, and water 33.5%) and Diet C for 4, 5 and 6 day-old larvae (royal jelly 50%, glucose 9%, fructose 9%, yeast extract 2%, and water 30%) (Ministry of Agriculture of the People’s Republic of China, 2017).

### 2.2 The honeybee

Ten healthy honeybee colonies were reared in the experimental apiary of the College of Animal Science (College of Bee Science), Fujian Agriculture and Forestry University (Fuzhou, China, and 26.08°N 119.23°E). Before the experiment, these colonies were not exposed to pesticides and the test larvae and pupae were obtained by the following method: Five healthy egg-laying queens were confined, respectively in empty combs for laying eggs within 8 h, and then these combs with new-laid eggs were moved to a separated place in the same colony. 3 days later, the 1-day-old larvae were swiftly transferred from the combs to the 96-well tissue culture plates by a Chinese grafting tool in the laboratory and kept in a dark incubator (Ningbo Jiangnan Instrument Factory) at 34°C ± 1°C, 95% ± 2% RH (Duan et al., 2021).

### 2.3 Fungicide treatment of *A. mellifera* larvae

Larvae in the plates were reared according to the method by Jensen et al. (2009) with a few modifications. Three tissue culture plates were taken as the control group, and the other six plates were taken as the two different concentration treatment groups. Three replicates per group and forty-eight larvae were treated per replicate. In the fungicide treatment groups, each larva was fed a contaminated diet containing different concentrations of pyraclostrobin, 1 day-old larvae were fed 20 μL Diet A containing fungicide, 3 day-old larvae were fed 20 μL Diet B including fungicide, and also 4, 5, and 6 day-old larvae were fed the Diet C with fungicide for 30, 40, and 50 μL, respectively. Meanwhile, the larvae in the control group were fed a normal diet with the same quantity as fungicide treatments (Ministry of Agriculture of the People’s Republic of China 2017; Dai et al., 2018). The artificial diet with fungicide was changed daily. The larvae normally pupate on the 7th day, so they were checked every 6 h before pupation, and the dead larva was removed and recorded. When they begin to emerge on the 19th day, the number of pupation and eclosion of larvae in each group was also recorded. The calculation method of capped rate and emergence rate was referred to Shi et al. (2020).

$$Capped\;rate=\frac{The\;number\;of\;capped\;cell}{total\;number\;of\;treated\;larvae}\times 100\%$$

$$Emergence\;rate=\frac{The\;number\;of\;emergence\;bee}{Total\;number\;of\;capped\;cell}\times 100\%$$

Fifteen white-eye pupae and fifteen newly emerged bees were randomly selected from each treatment and individually weighed to calculate the pupa weight and newly emerged bee birth weight. The newly emerged bee’s weight must be measured within 2 h after emergence. Moreover, ten 6-day-old larvae and ten pupae were randomly sampled from each treatment and immediately frozen with liquid nitrogen for RNA extraction.

### 2.4 Gene expression analysis

The total RNA of each individual was exacted by TRIzol^®^ Reagent (TransGen Biotech Co., Ltd. Beijing, China). After quality and concentration detection, the qualified RNA was used for cDNA synthesized by PrimeScript™ RT reagent Kit with gDNA Eraser (TaKaRa, Japan) and the cDNA samples were stored at −20°C. The qPCR assay was performed to examine the relative expression of development-related genes *Ecr* and *Usp*, nutrient metabolism-related genes *ILP1*, *ILP*2, *Hex70b*, *Hex110*, and *Vg*, and immune-related genes *Apidaecin*, *Abaecin*, *Defensin1* and *Hymenoptaecin* in larvae and pupae, respectively (Table 1). The *β-actin* was used as the reference gene (Duan et al., 2021) and the gene-specific primers were shown in Table 1. The qPCR was performed in ABI QuantStudio six Flex System (Thermo Fisher Scientific, Waltham, MA, and United States) with a 10 µL reaction volume containing TB Green Premix Ex Taq Ⅱ (2×) 5, cDNA 1 μL, each gene-specific primers (10 μM) 0.4 μL, ROX Reference Dye Ⅱ (50×) 0.2 μL and H_2_O 3 μL. The thermal procedure include 95°C for 30 s, followed by quantification for 40 cycles of denaturation at 95°C for 5 s, annealing at 60°C for 30 s, and a final melt-curve step was rung from 60°C–95°C for 10 s at 1°C increment to check for non-specific amplification. Both technical and biological triplicates were performed at least three in all experiments.

**TABLE 1:** Primers of development-, nutrient- and immune-related genes used for quantitative PCR.

Genes		Primer sequence(5′-3′)	Gene ID	Reference
Reference gene	*β-Actin*	F: TTG​TAT​GCC​AAC​ACT​GTC​CTT​T R: TGG​CGC​GAT​GAT​CTT​AAT​TT	NM_001185145.1	Simone et al. (2009)
Development-related genes	*Ecr*	F: GTT​TGC​GTT​TGG​AAA​GTC​ACG R: GGG​GGA​CCT​TTT​ATG​CGT​GT	XM_016913298.2	Liu et al. (2018)
	*Usp*	F: GGC​ACG​AGG​TAA​AAG​TGA​CGA R: TTA​GCC​AAG​TGT​TGC​CAC​GG	NM_001011634.2	
Nutrient-related genes	*ILP1*	F: TGG​CAA​GGT​GTC​TAT​CAC​CG R: ACG​TCA​GCA​GCA​TAT​CAC​CA	XM_026442143.1	De Azevedo and Hartfelder (2008)
	*ILP2*	F: TTC​CAG​AAA​TGG​AGA​TGG​ATG R: TAG​GAG​CGC​AAC​TCC​TCT​GT	NM_001177903.1	
	*Hex110*	F: ACA​ACA​AGC​AGG​ACA​ACA​GGA R: ACC​AAG​TCC​GTT​AGA​AAG​ACG​A	NM_001101023.1	Zheng et al. (2019)
	*Hex70b*	F: CCT​TGG​ACC​GAA​ATA​CGA​CGA R: GTG​TTG​CTT​CCG​CTT​TTC​AGG	NM_001011600.1	
	*Vg*	F: AGTTCCGACCGACGACGA R: TTCCCTCCCACGGAGTCC	NM_001011578.1	Simone et al. (2009)
Immune-related genes	*Abaecin*	F: CAG​CAT​TCG​CAT​ACG​TAC​CA R: GAC​CAG​GAA​ACG​TTG​GAA​AC	NM_001011617.1	
	*Apidaecin*	F: TTT​TGC​CTT​AGC​AAT​TCT​TGT​TG R: GTA​GGT​CGA​GTA​GGC​GGA​TCT	NM_001011613.1	Evans et al. (2006)
	*Defensin1*	F: TGC​GCT​GCT​AAC​TGT​CTC​AG R: AAT​GGC​ACT​TAA​CCG​AAA​CG	NM_001011616.2	
	*Hymenoptaecin*	F: CTC​TTC​TGT​GCC​GTT​GCA​TA R: GCG​TCT​CCT​GTC​ATT​CCA​TT	NM_001011615.1	

### 2.5 Statistical analysis

The Ct values of development-, nutrient-, and immune-related genes were normalized by the corresponding Ct value of reference gene *β*-actin, and then the relative expression levels of genes were calculated using the 2^−△△CT^ method (Livak and Schmittgen, 2001). All data are presented as mean ± standard error (S.E.). The one-way ANOVA was used to determine the significance of the differences in gene expression. With homogeneity of variance, the One-way analysis was followed by Tukey’s test. The significance level was set at a value of *p* < 0.05. All data analyses and figures were carried out using Graphpad Prism 8 (GraphPad Software, Inc., San Diego, CA, and United States).

## 3 Results

### 3.1 Pyraclostrobin exposure decreased the survival rate of larvae and pupae

The field concentrations of pyraclostrobin could repress the survival and development of *A. mellifera* larvae and pupae with noticeable toxic effects indicated by the significantly different survival and development index of *A. mellifera* larvae and pupae among fungicide treatments. The survival rate and capped rate of *A. mellifera* larvae from two pyraclostrobin treatments (83.3 and 100 mg/L) were significantly lower than that of the control groups (68.62%, 49.77% and 32.03%, 30.05%, *p* < 0.05, Table 2), and also the weight, emergence rate of pupae and newly emerged bee weight were also significantly decreased (*p* < 0.05, Table 3). Remarkably, there was a concentration-effect between treatment concentration and these development indexes, and a significant difference in the survival rate of larvae and birth weight of newly emerged bees between 83.3 and 100 mg/L treatment (*p* < 0.05).

**TABLE 2:** Effects of pyraclostrobin on the survival rate and capped rate of *Apis mellifera* larva.

Treatment	Survival rate (%)	Capped rate (%)
Control	97.21 ± 2.11 a	95.58 ± 1.61 a
83.3 mg/L	68.62 ± 5.86 b	32.03 ± 13.88 b
100 mg/L	49.77 ± 4.14 c	30.05 ± 7.49 b

Data in the table are mean ± SE (standard error) and the different letters mean significant difference (*p* < 0.05).

**TABLE 3:** Effects of pyraclostrobin on the pupa weight, emergence rate and newly emerged bee birth weight of *Apis mellifera*.

Treatment	Pupa weight (mg)	Emergence rate (%)	Birth weight (mg)
Control	202.68 ± 6.13 a	97.45 ± 0.33 a	154.88 ± 3.99 a
83.3 mg/L	174.02 ± 4.75 b	34.44 ± 15.03 b	123.97 ± 0.51 b
100 mg/L	159.98 ± 5.31 b	35.83 ± 15.07 b	102.00 ± 6.18 c

Data in the table are mean ± SE (standard error) and the different letters mean significant difference (*p* < 0.05).

### 3.2 Pyraclostrobin exposure interrupted the expression of development-related genes

**FIGURE 1:**
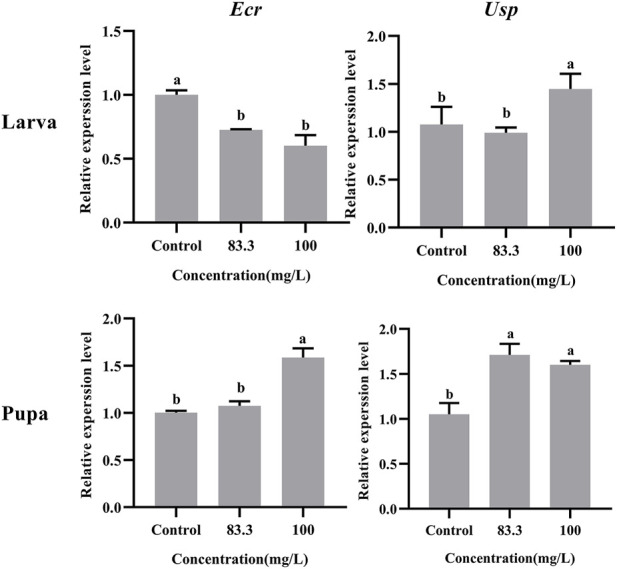
Effects of pyraclostrobin on the relative expression of development-related genes (*Ecr* and *Usp*) in *Apis mellifera* larva and pupa. The data in the figures are mean ± SE (standard error) and different letters above bars mean significant difference (*p* < 0.05, Fisher’s LSD test).

The relative expression of development-related genes (*Ecr* and *Usp*) in *A. mellifera* larvae and pupae was affected after exposure to field concentrations of pyraclostrobin (83.3 and 100 mg/L, Figure 1). The field concentrations of pyraclostrobin inhibited the expression level of *Ecr* in larvae and gradually downregulated with the increasing treatment concentrations (0.6-fold for 100 mg/L; 0.73-fold for 83.3 mg/L, *p* < 0.05). Meanwhile, the relative expression of *Usp* in larvae with 100 mg/L treatment was significantly upregulated than both the 83.3 mg/L treatment and control. However, the pyraclostrobin could induce the expression level of *Ecr* and *Usp* in pupae. The higher the treatment concentration, the stronger the induction effect. The expression level of *Usp* was significantly higher than in control (1.52-fold for 100 mg/L; 1.63-fold for 83.3 mg/L, *p* < 0.05), and the 100 mg/L treatment could significantly induce the expression level of *Usp* of pupae*.*

### 3.3 Pyraclostrobin exposure interfered with the nutrition metabolism of larvae and pupae

**FIGURE 2:**
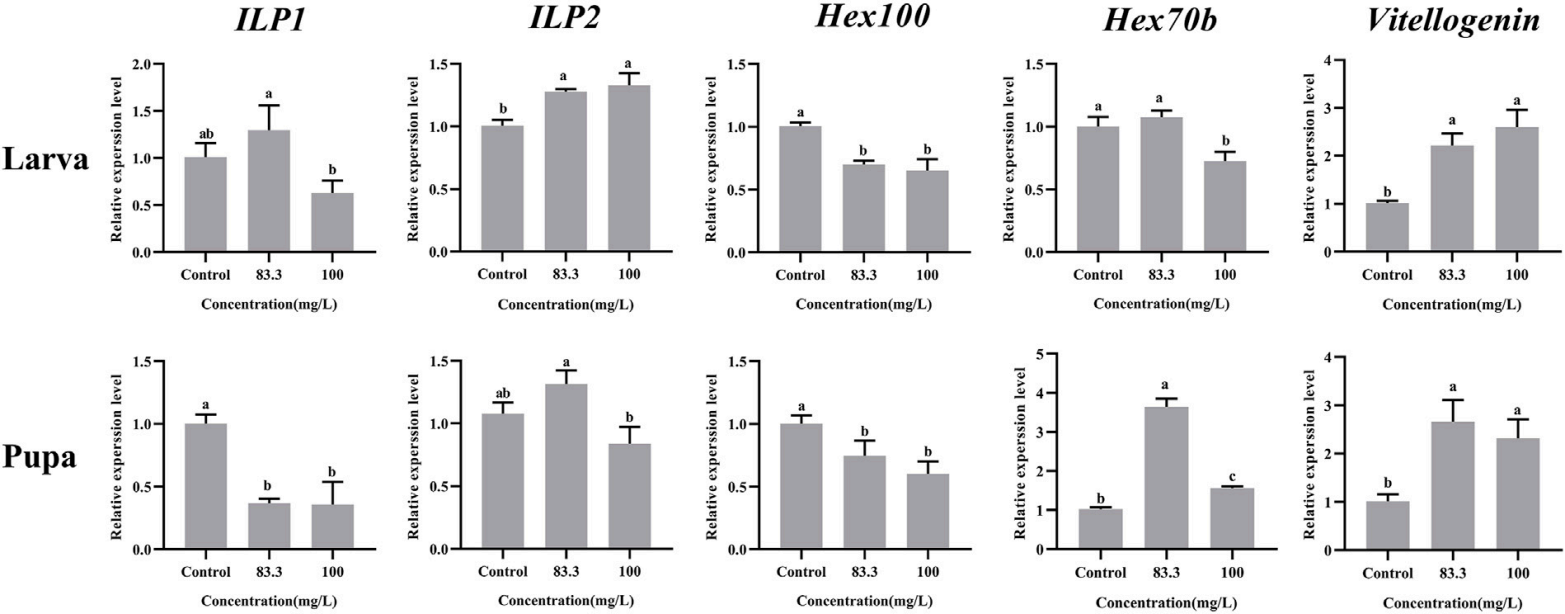
Effects of pyraclostrobin on the relative expression of nutrient-related genes (*ILP1*, *ILP2*, *Hex110*, *Hex70b*, and, *Vg*) in *Apis mellifera* larvae and pupae. The data in the figures are mean ± SE (standard error) and different letters above bars mean significant difference (*p* < 0.05, Fisher’s LSD test).

These five nutrient-related genes were all expressed in *A. mellifera* larvae and pupae but at varying levels under different pyraclostrobin concentrations (Figure 2). For larvae, the pyraclostrobin can significantly upregulate the expression of *ILP2* and *Vg*, but the expression of *Hex110* was downregulated in both treated concentrations (*p* < 0.05). And also the high concentration pyraclostrobin (100 mg/L) can significantly downregulate the expression of *Hex70b* in larvae. For pupae, the expression levels of *Hex70b* and *Vg* were significantly upregulated in two fungicide treatments (*p* < 0.05). Meanwhile, the expression levels of *ILP1* and *Hex110* were significantly downregulated with both pyraclostrobin treatments (*p* < 0.05). Despite the expression of both *ILP1* in larvae and *ILP2* in pupae being induced by low pyraclostrobin concentration (83.3 mg/L), which was also inhibited by high concentration treatment (100 mg/L), there were no statistical differences between the treatments and control.

### 3.4 Pyraclostrobin exposure disturbed the immunity of larvae and pupae

**FIGURE 3:**
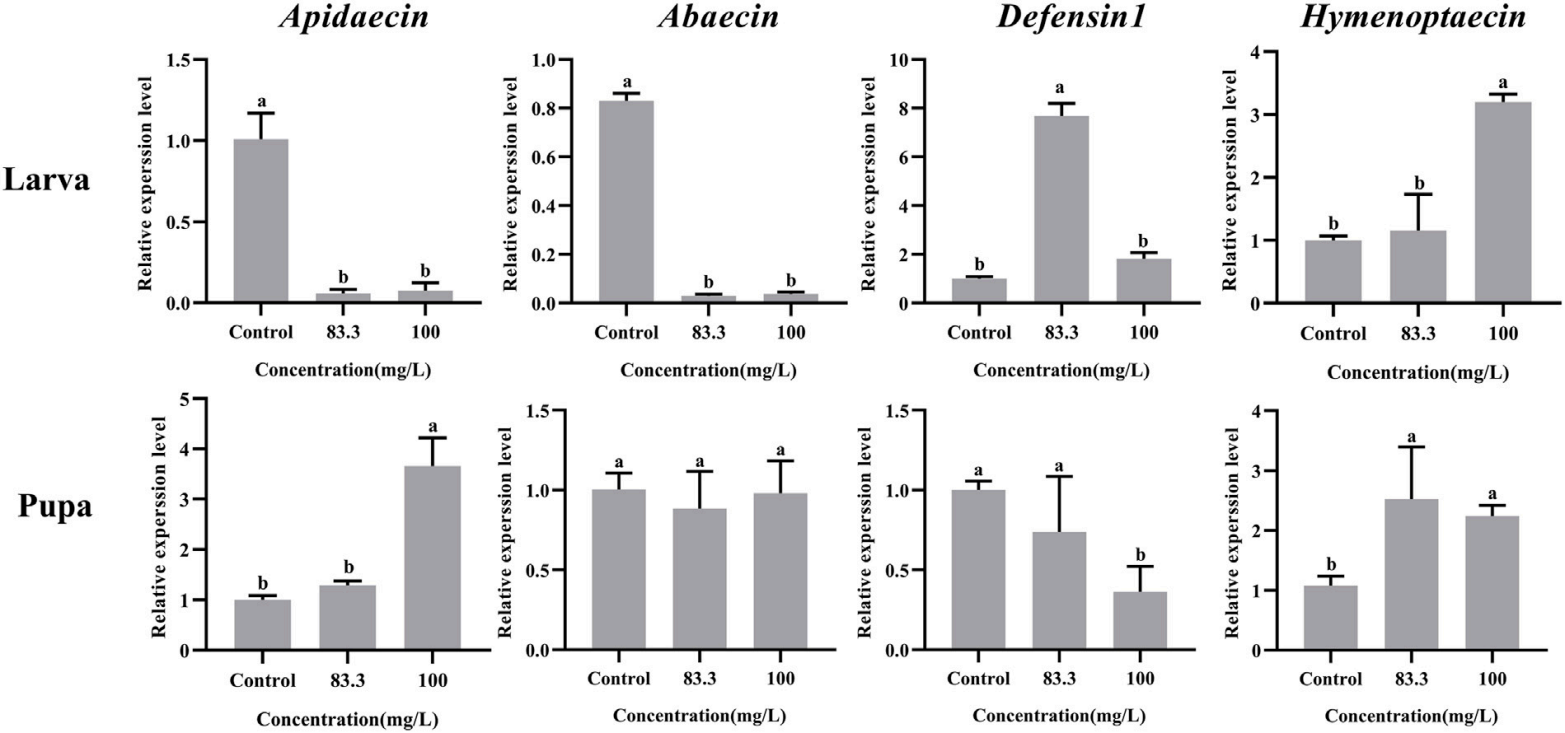
Effects of pyraclostrobin on the relative expression of immune-related genes (*Apidaecin*, *Abaecin*, *Defensin1,* and *Hymenoptaecin*) in *Apis mellifera* larvae and pupae. The data in the figures are mean ± SE (standard error) and different letters above bars mean significant difference (*p* < 0.05, Fisher’s LSD test).

The mRNA levels of these four immune-related genes in both larvae and pupae were influenced by pyraclostrobin exposure (Figure 3). For larvae, the expression of *Abaecin* and *Apidaecin* was significantly decreased in fungicide treatments (*p* < 0.05). However, the pyraclostrobin can induce the expression of *Defensin1* and *Hymenoptaecin*. The expression of *Defensin1* was significantly increased after exposure to 83.3 mg/L pyraclostrobin treatment, but the expression of *Hymenoptaecin* was significantly increased after exposure to high pyraclostrobin treatment (100 mg/L). For pupae, the expression level of *Apidaecin* and *Hymenoptaecin* was increased after exposure, and the expression of *Apidaecin* in 100 mg/L treatment was significantly higher than control. In addition, the expression of *Hymenoptaecin* was also significantly affected by pyraclostrobin. The expression of *Defensin1* was significantly decreased in fungicide treatments (*p* < 0.05). Although the expression of *Apidaecin* was lower than control, there was no significant difference (*p* > 0.05).

## 4 Discussion

So far, pyraclostrobin has been on the market for over 20 years and was registered and used for fungi diseases control of various plants in different countries (Luo et al., 2022). With widely and irrational use, the accumulation and pollution of pyraclostrobin in the soil, water and other ecosystems represent high potential risks to the environment and organisms. As an indicator of environmental pollution, honeybees also directly or indirectly contact with this pollutant which led to harmful effects on individuals and colonies (Yoder et al., 2013; David et al., 2015; Zaluski et al., 2017; Tadei et al., 2019; Xiong et al., 2022).

Despite the low acute oral and contact toxicity of pyraclostrobin to honeybees (Tan et al., 2021), long-time exposure to pyraclostrobin could lead to irreversible adverse effects on honeybees. The results showed pyraclostrobin (100 and 83.3 mg/L) were chronic toxicity effects on the survival and development of *A. mellifera*. da Costa Domingues et al. (2020) found the forager workers of *Melipona scutellaris* exposed to pyraclostrobin showed a reduced survival rate. Compared with control, both two pyraclostrobin concentrations exposure significantly reduced the survival rate of *A. mellifera* larvae with a significant concentration effect (*p* < 0.05; Table 2). Meanwhile, the significantly low capped rate of larvae and emergence rate of pupae after pyraclostrobin exposure causes unsuccessful metamorphic development from larvae to pupae with a high mortality rate. During the process of pupation and emergence, honeybees consume the energy which they have previously stored to synthesize new substances. Owing to the low concentration of pyraclostrobin could inhibit the mitochondrial respiratory of *A. mellifera* (Nicodemo et al., 2020), which means the fungicide exposure-treated *A. mellifera* larvae and pupae need to consume more material to complete metamorphosis. Thus significantly decreased the weight of pupae and newly emerged bees (Table 3), suggesting pyraclostrobin could affect the normal growth and metabolism of *A. mellifera* larvae and pupae, especially for pupae with a low emergence rate and weight, though they did not feed during pupal stage. The quantity and quality of brood (larvae and pupae) are critical to the population size of the colony (Duan et al., 2020b; Xiong et al., 2022) and these findings indicate pyraclostrobin can cause serious damage to bee colonies by suppressing the survival and development of individuals.

During the larval-pupal transition of *A. mellifera*, the development rhythm of metamorphosis was regulated by juvenile hormones (JH) and molting hormone (20-hydroxyecdysone, 20E) (Liu et al., 2014). The 20E, ecdysteroid receptor (*Ecr*) and ultraspiracle protein (*Usp*) constitute the ligand-receptor complex (20E-Ecr-Usp) and then activate the metamorphosis process (Riddiford et al., 2000). Therefore, the *Ecr* and *Usp* were considered to be key genes responsible for the transduction of the JH/20E signals during metamorphosis development (Barchuk et al., 2008; Duan et al., 2021). In the present study, the RT-PCR results showed the expression levels of *Ecr* and *Usp* in larvae and pupae were altered with pyraclostrobin exposure. The expression level of *Ecr* and *Usp* in pupae were significantly upregulated after pyraclostrobin exposure (100 and 83.3 mg/L). Furthermore, the low emergence rate and weight of pupae also confirmed that pyraclostrobin could disturb the normal development process leading to a high mortality rate of *A. mellifera* pupae.

Pyraclostrobin could cause the energy deficiency of *A. mellifera*, and more nutrition materials need to be metabolized for normal development (Nicodemo et al., 2020). Moreover, extra nutrient consumption may cause the weight loss of pupae and newly emerged bees. The present results suggested that the weight loss might come from either some nutrient metabolic pathway disturbance or the decreased hexamerins for building the pupae tissues, which may already be disrupted by pyraclostrobin. There are two insulin-like peptides (ILPs) in honeybees that have profound effects on invertebrate metabolism, nutrient storage and fertility (de Azeved and Hartfelder, 2008; Duan et al., 2021). The *ILP1* gene potentially functions in lipid and protein metabolism while *ILP2* is a more general indicator of nutritional status (Ihle et al., 2014). Compared with control, the high concentration of pyraclostrobin (100 m/L) inhibits *ILP1* expression in both larvae and pupae. However, the expression of *ILP2* in 83.3 mg/L treatment exhibited upregulation. The abnormal expression phenomenon of *ILP1* and *ILP2* in honeybees would lead to nutritional and metabolic disorders (Wang et al., 2013). The hexamerins were synthesized in the fat body during the larval growth phase and used for pupal development and adult differentiation (Burmester and Scheller, 1999). The subunits of *Hex 110* were highly abundant in *A. mellifera* larval hemolymph and the gene activity obeys a nutritional control (Bitondi et al., 2006). The expression of *Hex100* was significantly downregulated indicating malnutrition and developmental abnormalities of larvae and pupae after pyraclostrobin exposure. Meanwhile, the larvae could use *Hex 70b* to compensate for the lack of proteins (Cunha et al., 2005). And the expression of *Hex 70b* was significantly induced in pupae by pyraclostrobin (Figure 2). Vitellogenin (*Vg*) is an egg-yolk precursor in insect reproduction and multiple roles of Vgs, such as immunity, life span, and antioxidation in non-reproduction were also uncovered (Havukainen et al., 2013; Salmela et al., 2015; Salmela and Sundström, 2017). Pyraclostrobin exposure could result in oxidative stress in zebrafish embryos (Li et al., 2018; Kumar et al., 2020). In the present study, *Vg* has significantly upregulated expression in larvae and pupae which acts as a ‘defender’ against infection and reactive oxygen species for a prolonged life span (*p* < 0.05) (Havukainen et al., 2013; Salmela et al., 2015).

Honey bee innate immunity provides immediate responses against invading pathogens, especially antimicrobial peptides (AMPs) in cell-free humoral immunity (Danihlík et al., 2015; Duan et al., 2021; Xiong et al., 2022). Four families of AMPs (i.e., apidaecins, abaecin, hymenoptaecin and defensins) with a variety of antimicrobial activities have been described in the honey bee and their expressions were regulated by two intracellular signaling pathways Toll and Imd/JNK (Evans et al., 2006; Danihlík et al., 2015). It should be noted that that pyraclostrobin had negative effects on the immunity of bee larvae and pupae, leading to a low survival rate (Figure 3). At the larvae stage, the expression of *Apidaecin* and *Abaecin* were significantly downregulated (*p* < 0.05) indicating that exposure to pyraclostrobin makes the honeybee would be more sensitive to pathogens, which may be the main reason for the increased *Nosema ceranae* infection rates in adult bees (Pettis et al., 2013). While the *Defensin1* and *Hymenoptaecin* genes exhibited upregulation in two pyraclostrobin treatments suggesting that larvae can coordinate different immune genes in response to the effects of fungicides on their immunity (Shi et al., 2020). However, at the pupae stage, the pyraclostrobin exposure could induce the expression of *Apidaecin* and *Hymenoptaecin*, and inhibit the expression of *Defensin1* which means pupae had different defense strategies for stress to immunity than larvae. Furthermore, combined with the results of immune-genes expression with different exposure concentrations, these four immune genes have different response mechanisms to two pyraclostrobin treatment concentrations. Considering the regulation of four immune genes by Toll and Imd/JNK metabolic pathways (Evans et al., 2006), the influence of pyraclostrobin on two Toll and Imd/JNK metabolic pathways also needs to be further evaluated and attention.

## 5 Conclusion

In the current study, two field-recommended concentrations pyraclostrobin (100 and 83.3 mg/L) showed significant adverse effects on the development of honey bee, resulting in a significantly lower survival rate, capped rate, emergence rate and body weight. Meanwhile, with long-term pyraclostrobin exposure, the expression levels of development-, nutrient- and immune-related genes in both larvae and pupae were also abnormally altered, indicating that pyraclostrobin could impair the development, nutrient metabolism and immunity of larvae and pupae. These findings demonstrate that the low acute toxic fungicide pyraclostrobin has deleterious effects on *A. mellifera* larvae and pupae with continuous exposure. Thus, it is necessary to re-evaluate the safety and potential risks of the fungicides to honey bee, bumble bee and solitary bee in the future. And the health welfare of pollinators should be emphasized in integrated pest management.

## Data Availability

The original contributions presented in the study are included in the article/Supplementary Materials, further inquiries can be directed to the corresponding author.
